# Malaria Prevalence in Asymptomatic and Symptomatic Children Living in Rural, Semi-Urban and Urban Areas in Eastern Gabon

**DOI:** 10.1007/s11686-023-00783-x

**Published:** 2024-01-09

**Authors:** Lady Charlène Kouna, Sandrine Lydie Oyegue-Liabagui, Dominique Fatima Voumbo-Matoumona, Jean Bernard Lekana-Douki

**Affiliations:** 1 Unité d’Evolution, Epidémiologie et Résistances Parasitaires(UNEEREP), Centre international de Recherche Médicales de Franceville, Franceville, Gabon; 2 Ecole Doctorale Régionale d’Afrique Centrale en Infectiologie Tropicale, Franceville, Gabon; 3 Département de Biologie, Faculté des Sciences, Université des Sciences et Techniques de Masuku, Franceville, Gabon; 4 Département Masters/Licences, parcours types des sciences Biologiques, faculté des sciences et Techniques, Université Marien Ngouabi, Brazzaville, Congo; 5 Département de Parasitologie Mycologie et de Médecine Tropicale, Université des Science de la Santé, Libreville, Gabon

**Keywords:** Malaria, Asymptomatic, Symptomatic, Rural, Semi-rural, Urban

## Abstract

**Background:**

Malaria remains a major public health issue in the world despite a decline in the disease burden. However, though symptomatic malaria is diagnosed and treated, asymptomatic infections remain poorly known and support transmission. This study assessed the prevalence of symptomatic and asymptomatic *Plasmodium* spp. infections in three areas in Gabon to monitor and evaluate the impact of malaria.

**Methods and Results:**

A cross-sectional study was conducted in three areas of Gabon. Febrile and afebrile children aged 6 months to 15 years were included in this study. Malaria prevalence was determined by microscopy of and using rapid diagnostic test (RDT). *Plasmodium* spp. species were identified by PCR according to the Snounou method. The data were recorded in Excel, and the statistical analyses were performed using the software R version R 64 × 3.5.0. A total of 2381(333 asymptomatic and 107 symptomatic) children were included. The overall prevalence of malaria was 40% (952/2381), with the majority (77% symptomatic and 98% asymptomatic) of infections caused by *Plasmodium falciparum*. A high prevalence of malaria was found in infected children in rural and semi-rural areas. In these two areas, a higher prevalence of *Plasmodium malariae* was observed in asymptomatic. Furthermore, mixed infections were more prevalent in asymptomatic children than in symptomatic.

**Conclusion:**

This study showed that the prevalence of *Plasmodium* spp. infection varied according to the regions. The main species was *Plasmodium falciparum*, but in asymptomatic children the prevalence of *Plasmodium malariae* was high in rural areas. To help fight malaria more effectively asymptomatic infections should be taken into account and treated.

## Background

Despite a significant reduction in the burden of malaria infection in many countries over the past decades, malaria remains an important health issue in Africa. An estimated 228 million cases of malaria were recorded worldwide in 2018, compared to 239 million cases in 2010 [1]. Moreover, a recent World Health Organization report revealed that the estimates for the 2015–2017 period were similar which would suggest that the global malaria burden has been stabilized instead of reducing despite public health efforts. In 2016, an increase in the number of malaria cases was shown, with an estimated 216 million cases in 91 countries compared to 211 million cases recorded in 2015. An increase in the incidence of malaria was also reported in some areas [1]. Globally, the number of malaria deaths was estimated at 445 000 in 2016, compared with 446 000 the previous year [2]. The majority of malaria cases and deaths (93%) were reported by the WHO African region, mainly in children under five years of age [1].

Malaria is hyperendemic to Gabon, a country in Central Africa, and has perennial transmission [3]. The burden of the disease follows a seasonal pattern that is influenced by the equatorial climate which favors mosquito proliferation and larval development [4]. A total of 35 244 cases of malaria were reported in Gabon in 2017 [5]. Malaria incidence is also high and estimated at 97.5 per 1,000 people [6]. This is attributed to the poor implementation of control measures (percentage of use of long-lasting insecticide nets (LLINs): 27%; proportion of pregnant women who received intermittent preventive treatment (IPT): 12%), as well as the decrease in funding to fight malaria, and in preventive care in general [6].

In Gabon, the main *Plasmodium* species that causes malaria is *Plasmodium falciparum* [6], which accounts for 99.7% of deaths in the WHO African region (1). Two *Anopheles* species have been recorded as malaria vectors in Gabon: *Anopheles gambiae* and *Anopheles funestus* [6]. In addition, studies have shown that other species of *Plasmodium* including *Plasmodium malariae* and *Plasmodium ovale* are also present in Gabon. *P. falciparum* remains the main and the most virulent species, especially in urban areas, while the prevalence of other species remains very low [7–10].

In Gabon, malaria is the second leading cause of consultation and hospitalization in pediatric wards after respiratory tract infections. It is responsible for more than one-third of all febrile patients [7, 11, 12]. Almost all cases are due to *P. falciparum*, with prevalence rates ranging from 94 to 99%. Other *Plasmodium* species, mainly *P. malariae* (0.5-5%) and *P. ovale* (0.5–2.4%) have been found in the country at a relatively low prevalence [10, 13].

Data on the prevalence of *Plasmodium* infection in Gabon come mainly from clinical malaria. In 2016, *Plasmodium* spp. infection was 53 to 79% in rural areas (79.5% in Lastoursville and 53.6% in Fougamou), 21.2–36.1% in semi-urban and urban areas (36.1% in Koula-Moutou and 21.2% in Franceville) [14]. In Libreville, the capital of Gabon, the prevalence of *Plasmodium* spp. infection was 18.8% in 2018 [14]. However, little is known about the epidemiology of asymptomatic *Plasmodium* parasite infection in perennial transmission regions, especially in Gabon [15].

Studies characterizing and comparing asymptomatic *Plasmodium* parasite infection in children from rural and urban areas in Gabon are scarce [15]. Yet the prevalence of parasitemia in asymptomatic carriers remains an important issue because asymptomatic cases typically go undetected and untreated [16]. Thus, they constitute a hidden reservoir for the parasite that can contribute to the persistence of malaria transmission in both high- and low-transmission settings, potentially accounting for up to 90% of onward transmission by vectors [17–23]. Indeed, a positive correlation between transmission rates and prevalence of asymptomatic cases in regions exhibiting wide variation in overall disease prevalence has recently been shown in Nigeria, Senegal, Gabon, and Brazil [23]. Results from a previous study exploring the diversity of *Plasmodium* species infecting humans in Gabon showed that in asymptomatic children, the prevalence of *P. falciparum* (99.4%) and *P. malariae* (47.6%) are high compared to *P. ovale* (9.9%) [23]. Therefore, with the new initiative of the Global Malaria Programme « T3 - Test, Treat, Track », identifying asymptomatic *Plasmodium* parasite infection cases is critical to effectively target drug treatments. Moreover, detecting and treating both clinical malaria and asymptomatic *Plasmodium* spp. infection is essential to break the cycle of transmission in order to achieve malaria elimination. As such, this study investigated the distribution of different *Plasmodium* species in symptomatic and asymptomatic children in semi-rural and urban endemic areas in south-eastern Gabon.

## Methods

### Study Sites and Population

**Fig. 1:**
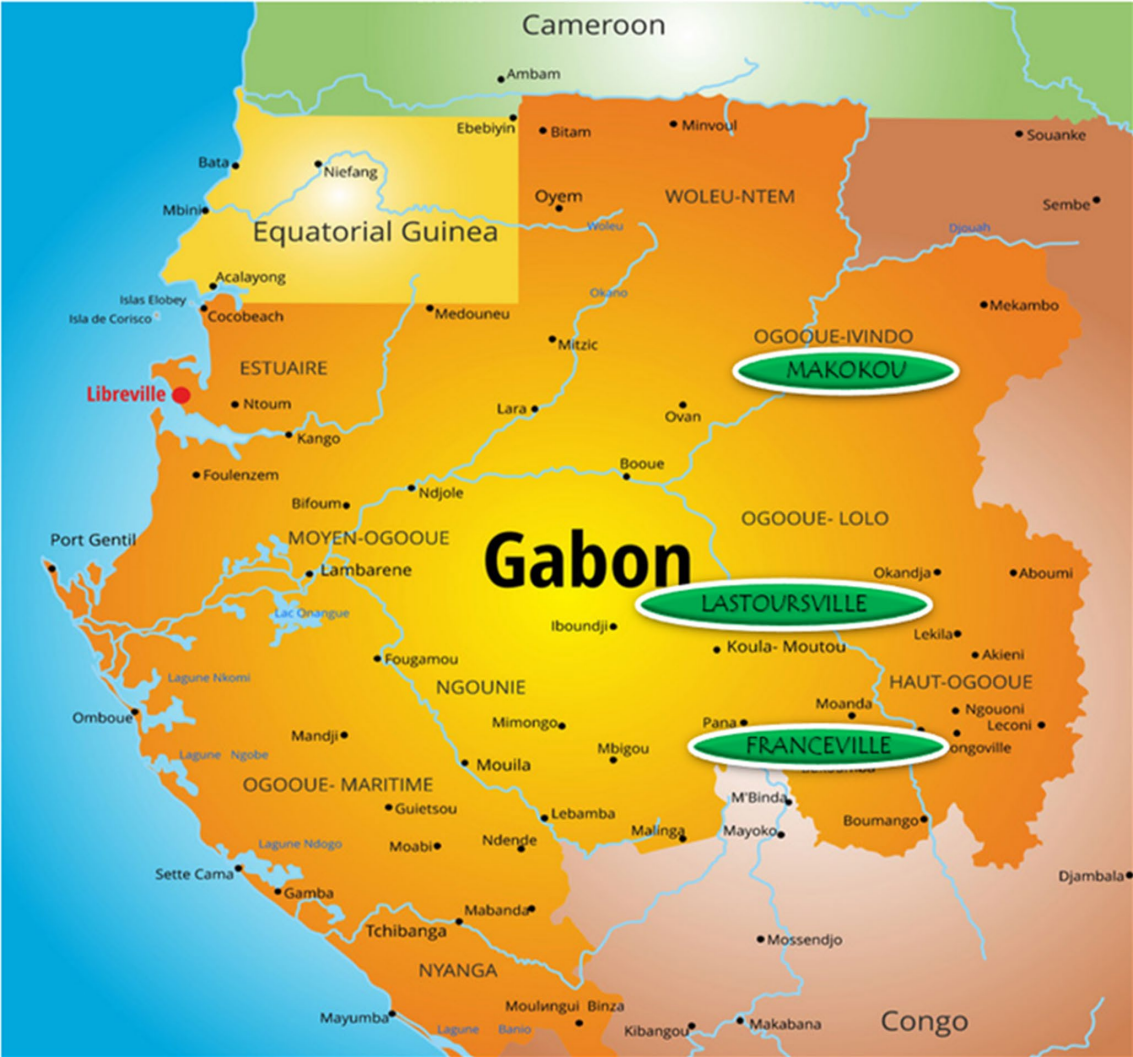
Map of Gabon showing the three sampling sites, Franceville (urban area), Makokou (semi-urban area) and Lastoursville (rural area)

A cross-sectional study was conducted between 2016 and 2018 in three areas of Gabon: Franceville, the administrative capital of the Haut-Ogooué Province and an urban area in South-Eastern Gabon (1°37′15″S and 13°34′58″E); Makokou, the administrative capital of the Ogooué-Ivindo Province and a semi-urban area in North-Eastern Gabon (0°33′33″N and 12°50′48″E); and Lastoursville, the capital of the Mulundu department and a rural region of Central-Eastern Gabon (0° 49′ S, 12° 42′ E) (Fig. 1). A total of 2381 children aged between 6 and 180 months (15 years) of whom 709 were symptomatic and 241 asymptomatic were enrolled in our study. The number of participants per school and per health centers was determined by their availability and willingness. All children were screened for *Plasmodium* infections and those positive by either rapid diagnostic tests (RDT) or microscopy were considered symptomatic or asymptomatic depending on the case. The symptomatic population was recruited in pediatric services of Hopital de l’Amitié Sino-Gabonaise in Franceville, Centre Hospitalier Régional Omar Bongo Ondimba in Makokou and Centre medical in Lastourville, and consisted of febrile children (≥ 37.5 °C) or with a history of fever less than 24 h before the consultation. The asymptomatic population was recruited in primary schools of Ombélé in Franceville, Notre Dame de Victoire A in Makokou, public schools of Matsatsa, Mana-Mana and Malende, in Mulundu department, and composed of children with a temperature < 37.5 °C who did not present a fever or history of fever during the seven days before enrollment.

For overall study sites, authorizations were obtained from administrative authorities, directors of provincial academy, regional health offices and heads of health centers. In school, following permissions, preliminary visits were made to school to present the study to head teachers. On the second visit, meetings were held at each school to explain purpose and procedures of the study to teachers and parents, followed by the distribution of written consent forms. Only children whose parents consented, and who also wanted to participate, were included in the study. Also, after obtaining written consent from parents/guardians, information related to gender, age, history of fever/malaria, treatment and body temperature, were recorded. In health facilities, samples were collected daily based on consultations at the pediatric services. Malaria-related symptoms and information on sex, age, and body temperature were recorded, followed by a physical examination by the physician after obtaining consent from the patients’ parents/guardians.

Both symptomatic and asymptomatic *Plasmodium* parasite infection cases were referred and offered appropriate treatment according to malaria treatment guidelines. Children who did not fill the criteria and those for whom the informed consent of parents or guardians was not obtained were excluded from the study. Laboratory screening and other routine procedures were conducted according to standards of care. (Fig. 1).

### Diagnosis of ***Plasmodium*** spp. Infection

The malaria diagnostics methods used were both RDT and microscopy. Malaria rapid diagnostic tests were performed using the Optimal-IT® RDT test to detect *P. falciparum* histidine-rich protein2, according to the manufacturer’s instructions. Thick blood smear microscopy slides were prepared according to the Lambaréné method as described in a previous study [24] and stained with a 20% Giemsa solution for 10 min. All blood smears were read by two different experienced technicians, and an internal quality control was performed by a third experienced reader for 10% of slides. The result is the arithmetic mean of the results found by the two technicians.

### Haematological Analysis

Routine hematological assays were performed with an automated haematology analyzer (STK, Coulter Corporation, USA). An amount of 5 ml of venous blood was collected in tubes containing ethylenediamine tetraacetic acid (EDTA) for the diagnostic of malaria. Blood elements were separated by centrifugation. Plasma was aliquoted and stored at − 80 °C until use and blood pellets were used for DNA extraction.

### DNA Extraction

DNA extraction was performed using the DNA Blood Omega Bio-tek E.Z.N.A® (Omega Bio-tek, Nor-cross, GA, USA) method according to the manufacturer’s protocol as previously described [8]. Briefly, 250 μl of blood, 25 μl of Omega Biotek (OB) protease (20 mg/ml), and 250 μl of lysis buffer were mixed and heated to 65 °C for 30 min before adding 260 μl of isopropanol. The mixture was transferred to a column and centrifuged at 10,000 rpm for 1 min. The column was washed twice at 13,000 rpm for 2 min, and DNA was eluted with 90 μL of sterile water preheated to 65 °C. DNA samples were kept at − 20 °C until use.

### Diagnosis of ***Plasmodium*** Species

Molecular diagnosis of *Plasmodium* species was done by PCR amplification according to the method described by Snounou [25]. The first PCR detects the *Plasmodium* genus using the primers rPLU5 (CCTGTTGTTGCCTTAAACTCC) and rPLU6 (TTAAAATTGTTGCAGTTAAAACG). The second PCR differentiates the species of *Plasmodium* by using 4 pairs of internal primers specific to the 4 *Plasmodium* species and whose sequences are shown in Table 1. Briefly, for the first PCR, five microliters of DNA were amplified with 1X buffer, 0.8 μM for each primer, 0.2 mM dNTP (Invitrogen®), 1.5 mM MgCl2 and 1 unit of Taq DNA polymerase (Invitrogen®) using the following cycling program: 1 min at 94 °C, then 30 cycles, 2 min at 60 °C, 2 min at 72 °C, and a final extension step of 7 min at 72 °C. The PCR was carried out in the Bio Rad® 4 °C thermocycler (Mercure de coanette, France). For the second PCR, two microliters of amplified DNA resulting from the first PCR were added in the reaction mixture and followed this cycling program: 30 s at 94 °C, then 30 cycles of 45 s at 45 °C, 1 min 30 s at 72 °C, and a final extension step of 7 min at 72 °C. PCR products were detected by 2% agarose gel electrophoresis. Only infected samples in blood smears were screened for *P. falciparum, P. vivax, P. ovale* and *P. malariae* (Table 1).

**Table 1:** Primers and PCR programs used to perform the detection of *Plasmodium* species

Species	Primers	Primer sequences (5’-3’)	Size a (pb)	Reaction	PCR conditions
*Plasmodium* spp.	rPLU5 rPlu6	CCTGTTGTTGCCTTAAACTTC TTAAAATTGTTGCAGTTAAAACG	1100	PCR1 (*P. falciparum*, *P. vivax*, and *P. malariae*)	Denaturation: 95 °C during 5 min and 94 °C during 1 min, hybridization at 60 °C during 2 min, and extension at 72 °C during 2 min (30 cycles)
*P. falciparum*	rFAL1 rFAL2	TTAAACTGGTTTGGGAAAACC AAATATATT ACACAATGAACTCAATCATGACTACCCGTC	205	PCR1 (*P. ovale*)	Denaturation: 95 °C during 5 min and 94 °C during 30 s, hybridization at 45 °C during 30 s, and extension at 72 °C during 1 min 30 s (30 cycles)
*P. vivax*	rVIV1 rVIV2	CGCTTCTAGCTTAATCCACAT AACTGATAC ACTTCCAAGCCGAAGCAAAGAAAGTCCTTA	120	Nested -PCR (*P. falciparum*, *P. vivax*, and *P. malariae*)	Denaturation: 95 °C during 5 min and 94 °C during 1 min, hybridization at 55 °C during 2 min, and extension at 72 °C during 2 min (30 cycles)
*P. ovale*	rOVA1 rOav2	ATCTCTTTTGCTATTTTTTAG TATTGGAGA GGAAAAGGACACATTAATTTGTATCCTAGTG	800	Nested- PCR (*P. ovale*)	Denaturation: 95 °C during 5 min and 94 °C during 30 s, hybridization at 45 °C during 30 s, and extension at 72 °C during 1 min 30 s (45 cycles)
*P. malariae*	rMAL1 rMal2	ATAACATAGTTGTACGTTAAG AATAACCGC AAAATTCCCATGCATAAAAAATTATACAAA	144		

### Statistical Analysis

The data collected were entered in an Excel spreadsheet, and then processed and transformed into a c.s.v or .txt file before imported into R software version R 64 × 3.5.0 for analysis (https://cran.r-project.org/bin/windows/base/old/3.5.0/R-3.5.0-win.exe). Age, hematologic parameters, and parasite densities were expressed as geometric means. Pearson’s χ^2^ test for categorical variables was used to compare group means. The non-parametric Kruskal–Wallis and Fisher’s exact tests were used to compare multiple groups of data. The Mann–Whitney U-non parametric test was used for pairwise comparisons. The statistical significance was set at *p* < 0.05.

### Ethical Statement

The study received ethical approval from the Gabonese National Research Ethics Committee (N°0023/2013/SG/CNE). Written informed consent was obtained from the parents or guardians before each child’s participation in the study.

## Results

### General Characteristics of the Study Population

The main characteristics of study participants recruited from health facilities as well as from schools are shown in Table 2. Participants were classified as having symptomatic or asymptomatic infections, microscopy positive, based on clinical diagnosis of parasitemia (via both microscopy and RDT) and the presence or absence of fever.

In the urban area, asymptomatic children were older (114.7 ± 43 months) than symptomatic children (64.3 ± 57 months; *P* < 0.05). White blood (6.3 ± 0.3 × 10^3^ cells/mm^3^) and red blood (3.99 ± 0.05 × 10^6^ cells/mm^3^) counts were lower in asymptomatic children with malaria than in symptomatic children (6.6 ± 0.4 × 10^3^ cells/mm^3^, 4.2 ± 0.1 × 10^6^ cells/mm^3^), but the difference was not statistically significant. Hemoglobin levels (9.74 ± 0.3 g/dl) and platelet counts (290 ± 16 × 10^3^ cells/mm^3^) were higher in symptomatic children than in asymptomatic microscopy positive children (9.3 ± 0.2 g/dl; 234 ± 10 × 10^3^ cells/mm^3^) but the difference was not significant.

In the semi-urban area, asymptomatic children were older (108.8 ± 61.3 months) than symptomatic children (40.61 ± 74.9 months; *P* < 0.05). Hemoglobin levels were significantly lower in symptomatic children (7.4 ± 0.12 g/dl) than in asymptomatic children (9.3 ± 0.2 g/dl; *P* = 0.000003). Red blood cell (3.34 ± 0.06 × 10^6^ cells/mm^3^) and platelet (213 ± 7 × 10^3^ cells/mm^3^) counts were also significantly lower in symptomatic children than in asymptomatic children (4.4 ± 0.2 × 10^6^ cells/mm^3^, 303 ± 18.6 × 10^3^ cells/mm^3^; *P* = 0.000002 and 0.0002, respectively). White blood cell counts (8.05 ± 0.6 × 10^3^ cells/mm^3^) were significantly lower in asymptomatic children than in symptomatic children (10.7 ± 0.4 × 10^3^ cells/mm^3^; *p* = 0.004).

In the rural area, asymptomatic children were older (84.7 ± 48.2 months) than symptomatic children (31.1 ± 58.9 months; *P* < 0.05), with a statistically significant sex ratio between the two groups (*P* = 0.007). In this locality, hematological parameters were analyzed only in symptomatic children. Mean values were 9.8 ± 0.4 × 10^3^ cells/mm^3^ for white blood cell counts, 3.1 ± 0.07 × 10^6^ cells/mm^3^ for red blood cell counts, 168.1 ± 9.4 × 10^3^ cells/mm^3^ for platelet counts and 7.2 ± 0.12 g/dl for hemoglobin levels.

Overall, there was a significant difference regarding hemoglobin levels and platelet, red blood cell and white blood cell counts between the groups of symptomatic and asymptomatic children from the three localities. In asymptomatic children, only white blood cell and platelet counts were significantly different in two localities. The mean age was significantly higher in asymptomatic children than in symptomatic children in the three localities (*P* < 0.05). The average age was significantly different between the urban, rural and semi-rural environments, in symptomatic and asymptomatic children.

**Table 2:** General characteristics of the children population

Study sites	Rural area			Semi-urban area			Urban area			P*	P**
Clinical status	ASYMP (N = 476)	SYMP (N = 401)	*P*	ASYMP (N = 129)	SYMP (N = 496)	*P*	ASYMP (N = 143)	SYMP (N = 737)	*P*		
Age (month ± SD)	84.7 ± 48.2	31.1 ± 58.9	< 0.05	108.8 ± 61.3	40.61 ± 74.9	< 0.05	114.7 ± 43	64.3 ± 57	< 0.05	1.46e-14	< 0.05
Sex ratio	0.8	1.3	0.007	1.8	1.1	0.3	1	1.3	0.6	0.7	0.2
Temperature (°C)	36.4 ± 1.4	37.6 ± 1.06	< 0.05	36.8 ± 3.3	38.8 ± 0.5	0.008	36.6 ± 0.9	37.5 ± 0.5	< 0.05	< 0.05	0.06
White blood cells (10^3^/mm^3^ ± SE)	ND	9.8 ± 0 0.4		8.05 ± 0.6	10.7 ± 0.4	0.004	6.6 ± 0.4	6.3 ± 0.3	0.7	0.06	< 0000
Red blood cells (10^6^/mm^3^ ± SE)	ND	3.1 ± 0.07		4.4 ± 0.2	3.34 ± 0.06	0.000002	4.2 ± 0.1	3.99 ± 0.05	0.1	0.3	< 0000
Haemoglobin (g/dl ± SE)	ND	7.2 ± 0.1		9.3 ± 0.2	7.4 ± 0.12	0.000003	9.3 ± 0.2	9.74 ± 0.3	0.2	0.3	< 0000
Platelets (10^3^/mm^3^ ± SE)	ND	168.1 ± 9.4		303 ± 18.6	213 ± 7	0.0002	234 ± 10	290 ± 16	0.3	0.002	< 0000

Age, sex ratio, temperature, hemoglobin level, platelets, white and red blood cell in symptomatic and asymptomatic children infected with Plasmodium parasite in all study sites (Lastoursville, Makokou, Franceville). Asymp: asymptomatic children; Symp: symptomatic children. *P*: *p*-value between asymptomatic and symptomatic children in each site; P*: *p*-value between asymptomatic children from all sites; P**: *p*-value between symptomatic children from all sites; ND: not determined. SE: Standard error. Statistically significant differences between two and three groups were tested using Mann–Whitney and Kruskal-Wallis methods respectively. Any *p* < 0.05 was considered statistically significant

### Demographical Characteristic of Infected Children

A total of 2381 participants were enrolled in the study. The children were aged 6 to 180 months, with a mean age of 64 ± 1.5 months. Among these, 1634 consulted the pediatric service and 747 were children from schools. For determination of the prevalence of *Plasmodium* spp. infection only microscopy and RDT were used.

The mean age was significantly higher in asymptomatic children than in symptomatic children in Franceville (symptomatic: 75.18 ± 3.6 months; asymptomatic: 107.5 ± 5.1 months) (*P* = 0.001). In the semi-urban area, the analysis of demographic parameters reveals a high average age in asymptomatic children (113 ± 8 months) compared to symptomatic children (44 ± 2 months) (*P* = 0.0001). In the rural area of Lastoursville, the mean age of asymptomatic children was higher (59 ± 2 months) than that of symptomatic children (34.01 ± 2 months) (*P* = 0.03) (Table 3).

The mean parasitemia was higher in symptomatic children with malaria (6691 ± 816 parasites/μl) than in asymptomatic children (4364 ± 1494 parasites/μl) in urban area, but the difference was not statistically significant. In semi-urban area, mean parasitemia was significantly higher in symptomatic children (46,270 ± 9002 parasites/μl) than in asymptomatic children (17,427 ± 4868 parasites/μl) (*P* = 0.006). In rural area, parasite burden was calculated only in asymptomatic children, and was 17,248 ± 3521 parasites/μl (Table 3).

**Table 3:** Parasite density and demographical characteristics of infected children

Study sites	Rural area			Semi-urban area			Urban area			P*	P**
Parameters	ASYMP (N = 201)	SYMP (N = 248)	*P*	ASYMP (N = 22)	SYMP (N = 294)	*P*	ASYMP (N = 23)	SYMP (N = 167)	*P*		
GMPD	17,248 ± 3521	ND		17,427 ± 4868	46,270 ± 9002	0.006	4364 ± 1494	6691 ± 816	0.3	0.8	< 0.05
Age (month ± SD)	59 ± 2	34.01 ± 2	0.003	113 ± 8	44 ± 2	0.0001	107.5 ± 5.1	75.18 ± 3.6	0.001	< 0.05	< 0.05
Sex ratio	0.7	1	0.3	1.5	1	0.3	1	1.2	> 0.05		

Geometric mean parasite density, age and sex ratio in symptomatic and asymptomatic children infected with Plasmodium parasite in all study sites (Rural area, Semi urban area, and urban area). Asymp: asymptomatic children; Symp: symptomatic children. *P*: *p*-value for symptomatic and asymptomatic children for each locality, P*: *p*-value for asymptotic individuals for the 3 localities, GMPD: geometric mean parasite density; P**: *p*-value of symptomatic individuals for the 3 localities; SD : Standard deviation. Statistically significant differences between two and three groups were tested using Mann–Whitney and Kruskal-Wallis methods respectively. Any *p* < 0.05 was considered statistically significant; ND: not determined

### Global Prevalence of *Plasmodium* spp. by Microscopy and RDT

**Fig. 2:**
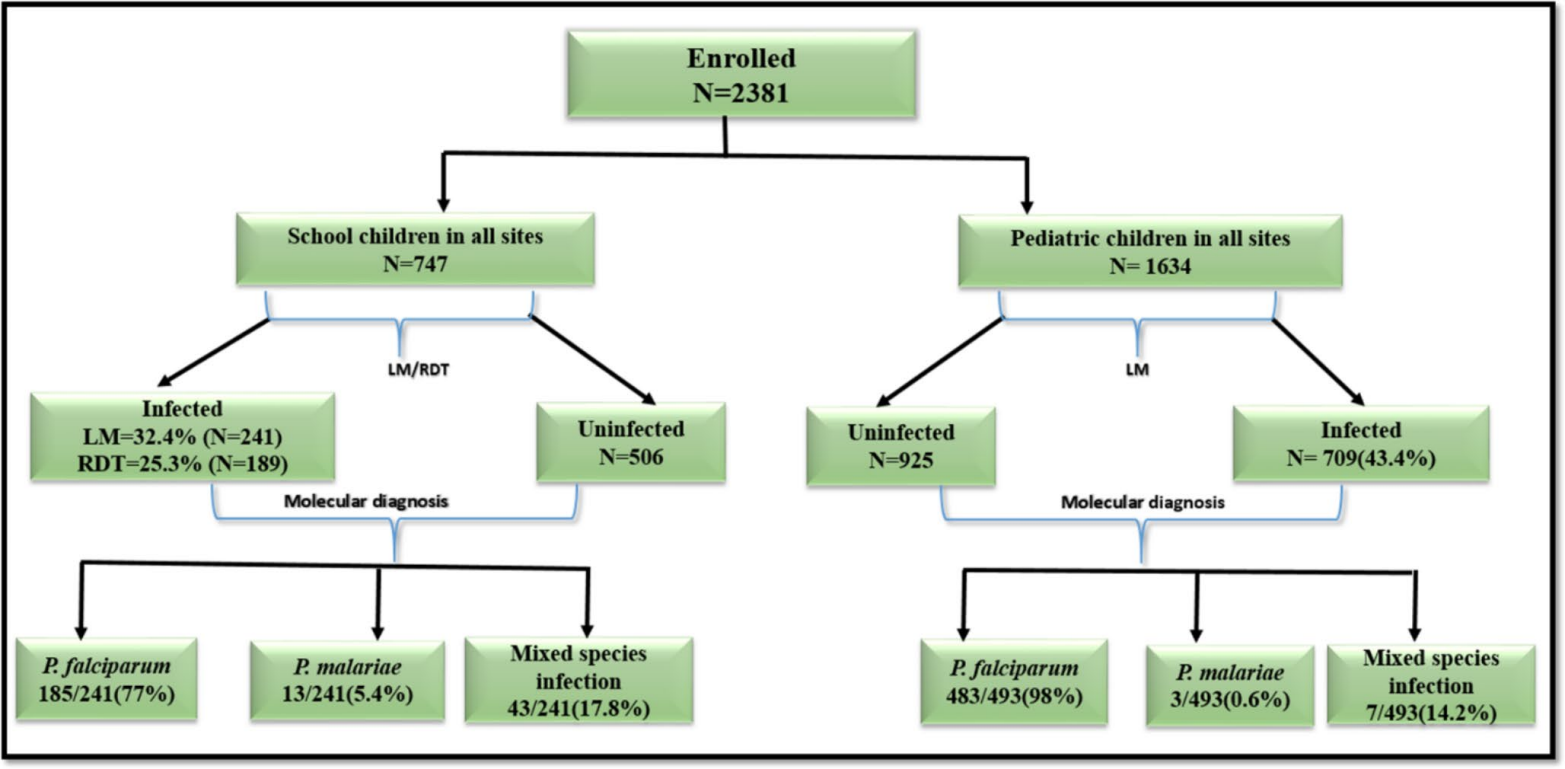
Distribution and prevalence of *Plasmodium* infection among the study participants

The Overall, prevalence of *Plasmodium* spp. was 32.3% (241/747) and 25.3% (189/747) in asymptomatic children by microscopy and RDT respectively. The prevalence of *Plasmodium* spp. was 43.2% (709/1634) in symptomatic children by microscopy. The overall prevalence of *Plasmodium* spp. infection determined by microscopy was significantly higher in symptomatic children than in asymptomatic children, (*p* < 0.05). (Fig. 2)

### Prevalence of ***Plasmodium*** Species by PCR

The determination of the *Plasmodium* species was only carried out by the PCR method in the samples diagnosed as positive by microscopy. Also, the *Plasmodium* species identified were *P. falciparum, P. malariae and P. ovale.* Global prevalence of malaria parasites was 91.4%, 2.8% and 6.2% for *P. falciparum*, *P. malariae* and mixed infections, respectively. In the overall study population, *P. falciparum* was significantly more prevalent than the other species (*P* < 0.05). Among the 241 asymptomatic participants, the highest prevalence recorded was for *P. falciparum (*77%: 185/241), followed by mixed (17.8%: 43/241) and *P. malaria* (5%: 13/241) infections (*P* < 0.05). Similar results were found in symptomatic children with 98% (483/493), 14.2% (7 /493) and 0.6% (3/493) for *P. falciparum*, mixed and *P. malariae* infections, respectively.

The prevalence of *P. falciparum* was significantly higher in symptomatic children (98%: n = 483/493) than in asymptomatic children (77%: n = 185/241; *P* = 0.03). In contrast, the prevalence of *P. malariae* infection was significantly higher in the asymptomatic children (5.4%: n = 13/241) than in the symptomatic children (0.6%: n = 3/493; *P* = 0.001). The prevalence of mixed *P. falciparum* + *P. malariae* infections was significantly higher in microscopy positive asymptomatic children (17.4%: n = 42/241) compared to symptomatic children (1.4%: n = 7/493, *P < 0.05*) (Fig. 2).

### Comparison of ***Plasmodium*** spp. Infection Prevalence Between Symptomatic and Asymptomatic Children in Urban, Semi-Urban and Rural Areas by Microscopy

Among 950 malaria positive children, 190 (23 microscopy positive asymptomatic and 167 symptomatic) resided in the urban area, 449 (201 asymptomatic and 248 symptomatic) in the rural area and 316 (294 asymptomatic and 22 symptomatic) in the semi-rural setting.

In the urban area, the prevalence of global *Plasmodium* spp. infection was 21.6% (n = 190/880). Although no significant difference in prevalence was observed between the two study groups, the prevalence of symptomatic *Plasmodium* spp. infection was 22.7% (n = 167/737) and that of asymptomatic infection was 16.1% (n = 23/143; Table 4). In semi-rural area, the overall prevalence of *Plasmodium* spp. was 50.6% (n = 316/625). In this setting, the prevalence of *Plasmodium* spp. infections was significantly higher in symptomatic (59.3%: n = 294/496) than in microscopy positive asymptomatic children (17%: n = 22/129; *P* < 0.05; Table 4).

In rural area, the overall prevalence of *Plasmodium* spp. was 51.2% (n = 449/876). In this locality, the prevalence of *Plasmodium* spp. was higher in microscopy positive asymptomatic infections (42.3%, n = 201/475) than in symptomatic infections (61.9%, n = 248/401). (*P* = 0.002; Table 4).

**Table 4:** Prevalence and distribution of *Plasmodium* spp

Study sites	Rural area			Semi-urban area			Urban area			P*	P**
Clinical status	ASYMP (N = 475)	SYMP (N = 401)	*P*	ASYMP (N = 129)	SYMP (N = 496)	*P*	ASYMP (N = 143)	SYMP (N = 737)	*P*		
LM	42.3% (201)	61.9% (248)	*0.002*	22 (17%)	294 (59.3% )	< 0.05	23 (16.1%)	167 (22.7%)	> 0.05	< 0.05	< 0.05
*P. falciparum*	151 (75.12%)	119 (99.1%)	*0.1*	17 (85%)	231 (97.3%)	*0.7*	17 (86.9%)	133 (97.09%)	*0.7*	*0.9*	*0.9*
*P. malariae*	10 (6.02%)	0	*0.01*	3 (15%)	3 (1.3%)	*0.009*	0	0		*0.07*	*0.2*
*Co-infection* *(P. falparum/* *P. malariae)*	40 (24.09%)	1 (0.8%)	< 0.05	0	2 (0.8%)	*1*	2 (8.7%)	4 (2.9%)	*0.2*	*0.2*	*0.2*
*Co-infection* *(P. falciparum/* *P. malariae/P. ovale)*	0	0		0	0		0	1 (4.35%)	*0.1*		*0.3*

Prevalence of different types of *Plasmodium* found in symptomatic and asymptomatic children from the three study areas (Rural, Semi-urban and urban areas). RDT: rapid diagnostic test; LM: light microscopy; Asymp: asymptomatic children; Symp: symptomatic children; *P*: *p*-value between asymptomatic and symptomatic children; P*: *p*-value between asymptomatic children from all sites; P**: *p*-value between symptomatic children from all sites. Statistically significant differences between two and three groups were tested using Mann–Whitney and Kruskal-Wallis respectively. Any *p* < 0.05 was considered statistically significant

### Comparison of ***Plasmodium*** Species Prevalence Between Symptomatic and Asymptomatic Children in Three Localities by PCR

To determine the distribution of *Plasmodium* spp. species, the samples from symptomatic and asymptomatic children, were analyzed by conventional PCR. The prevalence of the different *Plasmodium* parasites species was carried out on a total of children, among 160 (137 asymptomatic microscopy positive and 23 symptomatic) resided in the urban area, 32 (201 microscopy positive asymptomatic and 120 symptomatic) resided in the rural area and 256 (20 microscopy positive asymptomatic and 236 symptomatic) resided in the semi-urban area.

In urban area, 97.08% (n = 133/137) of symptomatic children were mono-infected with *P. falciparum* and 2.9% (n = 4/137) of the samples were coinfected with *P. falciparum* and *P. malariae*. In asymptomatic children, the prevalence of *P. falciparum* monoinfection was 73.9% (n = 17/23), and the percentage of co-infection with *P. falciparum* and *P. malariae* was 8.7% (n = 2/23). A triple infection with *P. falciparum* + *P. ovale* + *P. malariae* was also observed in one child (4.35%, n = 1/23). No significant difference in the distribution of *Plasmodium* species between symptomatic and asymptomatic children was observed (Table 4).

In rural area, the prevalence of *P. falciparum* was higher in symptomatic patients (99.1%, n = 119/120) than asymptomatic children (75.12%, n = 151/201) (*P = 0.002).*With regard to *P. malariae*, the prevalence of monoinfection in asymptomatic children was 5% (n = 10/201), suggesting a high prevalence of *P. malariae* in asymptomatic rural patients. Only *P. falciparum* + *P. malariae* mixed-infections were found with a prevalence of 0.8% (n = 1/120) and 19.9% (n = 40/201) in symptomatic and asymptomatic, children respectively, with a significant difference between the two groups (*P* < 0.05; Table 4).

The prevalence of *P. falciparum* symptomatic children (97.9%, n = 231/236) was significantly higher compared to the asymptomatic children (85%, n = 17/20) (*P* < 0.05). The prevalence of *P. malariae* was significantly lower in symptomatic children (1.3%: n = 3/236) compared to the asymptomatic children (15%: n = 3/20, *P* < 0.05). *P. falciparum* + *P. malariae* mixed-infections were found only in symptomatic children with a prevalence of 0.8% (n = 2/236; Table 4).

### Comparison of ***Plasmodium*** spp. Infections Between Asymptomatic and Symptomatic Populations Depending on the Area

The prevalence of *Plasmodium* spp. infections was significantly different among the symptomatic children in the three localities. Indeed, a significantly higher prevalence of infection was observed in symptomatic children in the rural and semi-urban areas compared to the urban area (*p* < 0.05). On the other hand, among asymptomatic children, the prevalence of *Plasmodium* spp. infections was significantly higher in the rural area compared to the urban and semi-urban areas (*p* < 0.05; Table 4).

Overall, *P. falciparum* was the most predominant species in the three localities, followed by *P. malariae*. No significant difference in species was observed in all three localities either in symptomatic or asymptomatic children. A single case of mixed *P. falciparum* + *P. malariae* + *P. ovale* infection was found in an asymptomatic child in Franceville. Furthermore, no *P. vivax* and *P. knowlesi* infections were observed in the study population (Table 4).

## Discussion

This study on the epidemiology of malaria in Gabonese children is the first to assess the prevalence of *Plasmodium* spp. infection between symptomatic and asymptomatic children in Gabon, in three localities with different epidemiological contexts.

In this study, the average age of asymptomatic children was significantly higher than that of symptomatic children in the three localities. These data are consistent with the results of previous studies which have shown that asymptomatic children are older than symptomatic children [16, 26]. This could be explained by the fact that the study was carried out in malaria-endemic areas. As such, age is an important determinant of protection against clinical malaria in endemic areas. Indeed, young children are more vulnerable, but adults and older children who have acquiered a form of immunity after cumulative exposure to the parasite are more likely to carry asymptomatic infections [16, 26]. The haematological parameters varied considerably depending on the symptomatology and the socio-geographic factors. A decrease in the hemoglobin level was observed in all infected children and induced anemia in symptomatic patients, this is confirmed by the low average number of red blood cells of infected children. Although the etiology of anemia in tropical areas is multi-factorial, our data are consistent with several other studies showing that anemia during *P. falciparum* malaria is closely associated with malaria parasitemia [27–29]. Similarly, it has been shown that in children infected with *P. falciparum* a combination of hemolysis of parasitized and non-parasitized red blood cells and erythropoiesis depression inducing anemia are often observed [30]. In contrast, in Franceville, the levels of red blood cells and hemoglobin were not significantly different between symptomatic and asymptomatic children. This may be due to the fact that in this locality the parasite densities were not significantly different between the two groups of children, since it was shown that parasite density has an impact on the occurrence of anemia in an individual [30]. Furthermore, in the semi-rural area, although a decrease in the average hemoglobin level and in the number of red blood cells was observed in the two groups, these two haematological parameters were significantly low in symptomatic children compared to asymptomatic children. This difference could be due to a massive destruction of red blood cells in symptomatic children in this locality. The average high parasite densities observed in symptomatic children could also explain this difference [30]. Moreover, the high prevalence of anemia in rural areas could be due to the high circulation of intestinal parasites in these areas [14]. Although thrombocytopenia in infected children in sub-Saharan African regions is very common [30], our study did not find the presence of thrombocytopenia in the children. In the group of symptomatic children, only the red blood cell and the hemoglobin levels were significantly different between the three localities. A difference in the white blood cell count and the number of platelets was observed in the group of symptomatic children. These results are consistent with the results of previous studies carried out in different regions of Gabon which have shown similar profiles in terms of hematological parameters according to the living area of infected children [8, 31]. We assessed the prevalence of *Plasmodium* spp. parasites infection in febrile and non-febrile children in urban, semi-urban and rural settings. The data presented here highlight a large difference in the prevalence of *Plasmodium* spp. l infection in the three localities. This makes it possible to confirm and understand the disparity in the epidemiological characteristics of *Plasmodium* spp. infection in Gabon. The global and symptomatic prevalence of *Plasmodium* spp. infection was higher in Lastoursville (rural area) and Makokou (semi-urban area) than in Franceville, as shown in a recent study in symptomatic patients [31]. Thus, the difference in malaria prevalence between the three areas could be due to better management of children aged under 5 years old by anti-malarial programs in Gabon and on the other hand, to a poor access to malaria control measures in rural and semi-urban areas. This difference in malaria prevalence could also be explained by the socio-economic level [31] and heterogeneity of transmission in Gabon, which have been demonstrated in other endemic areas [32]. This heterogeneity of transmission was also observed in the town of Libreville in Gabon, where the level of urbanization, the type of housing and the socio-economic level have an impact on the transmission of parasite [12]. On the other hand, urban environments are less favorable for vector species, in particular *An. gambiae*, which has a strong preference for unpolluted waters [33]. The lifespan of *An. gambiae* in urban areas was estimated to be less than half its lifespan in rural areas (4.1 versus 11 days) in a study in Kinshasa, the capital of the Democratic Republic of the Congo [34]. Mosquito dispersal is also much more limited in urban areas due to the higher housing density [35], which focuses the transmission of urban malaria [36]. In Franceville, people live in better housing than in rural and semi-urban areas. Better housing reduces the risk of malaria because it minimizes mosquito entry points during the night. To illustrate this, a study in Gambia has shown that homes with children infected with malaria are more likely to have mud walls, open eaves and absent ceilings than those with uninfected children [37].

In urban areas, the overall prevalence of *Plasmodium* spp. infection was estimated at 21.6%. These results are in agreement with those of the previous studies carried out in Franceville which showed that the prevalence of *Plasmodium* parasite infection in symptomatic children was approximately 20% [7, 31]. The lack of difference in prevalence between asymptomatic and symptomatic children confirms the low level of circulation of the parasite in this locality. Similarly, the low prevalence of *Plasmodium* infection in urban areas is related to the lower number of mosquitoes per person in these areas compared to rural areas. As a result, immunity takes longer to develop in urban areas, hence the higher average age of symptomatic children in urban areas. Also, the level of prevalence in asymptomatic children could explain the stagnation in the prevalence of malaria since 2010 despite the implementation of infection control policies in Gabon [8, 9, 31].

The overall prevalence of *Plasmodium* spp. infection in semi-urban and rural areas was comparable to that described above [31, 38]. The proportion of febrile children with *Plasmodium* spp. infection in semi-urban and rural areas was significantly higher than that of non-febrile children. This observation may be due to the fact that in these two localities the mean age varied significantly between the infected children, since it has been shown that asymptomatic infections are more frequent in young adolescents and adults residing in urban areas with endemic malaria. Because young adolescents and adults have acquired protective immunity through exposure to the parasite, it would limit the onset of symptoms. This is not the case for children under five years old who are at risk of developing malaria [39]. These results are not in agreement with the results of a study carried out in Cameroon in a semi-urban environment which reports a significantly high prevalence in asymptomatic children (30.7%) compared to symptomatic patients (17.8%) [40]. The prevalence of asymptomatic infections recorded in these 2 environments remains significant, since people with asymptomatic *Plasmodium* spp. infections are silent reservoirs of the parasite and pose a serious challenge to malaria health efforts because of their ability to maintain transmission among the population.

It was also observed in this study that the prevalence of asymptomatic *Plasmodium* spp. infection was high in rural areas compared to the two other localities. This observation is supported by the fact that children in rural areas have developed non-sterilizing anti-malarial immunity due to a higher exposure to the parasite. This is not the case with children in urban areas who are overprotected. Therefore, the fact that the prevalence of asymptomatic infections is high in rural areas ensures an increased maintenance of the prevalence of *Plasmodium* spp. infection in these localities. This study shows that the prevalence of malaria in Gabon differs considerably depending on the local economic status, confirming previous data [38]. This can be explained by the fact that in rural areas, antimalarial drugs are misused despite the availability of artemisinin-based combination therapy (ACT). Poor socio-economic conditions and insufficient knowledge of malaria could contribute to the high prevalence of malaria in rural areas, as noted in a recent study [31].

We also carried out molecular analyzes based on Snounou’s method [25] in order to determine the *Plasmodium* species circulating. Our analysis made it possible to highlight three *Plasmodium* species (*P. falciparum*, *P. malariae*, *P. ovale*) and to determine their prevalence in terms of mono- and co-infections. In general, the species were determined for 712 samples, representing a success rate of 91.4%. *P. falciparum* was responsible for the majority of *Plasmodium* spp. infections with an overall prevalence of 91.4%. This can be explained by the fact that *P. falciparum* is the main species in Gabon. Indeed, our results are consistent with our previous studies carried out in Gabon, which already showed that *P. falciparum* infections were the most widespread in urban, semi-urban and rural areas, whether in symptomatic or asymptomatic individuals [7, 10, 13]. This suggests that, despite the reduction in the burden of malaria in Gabon, the distribution of species among infected children has not changed and that *P. falciparum* remains the main species involved. In contrast, among asymptomatic people living in rural Gabon, it has recently been reported by two studies that *P. malariae* accounted for 21.9–47.6% of cases of p *Plasmodium* spp. Infections [23, 41]. These results are consistent with those of our study which classify this species in second position after *P. falciparum* in *the* semi-urban and rural environments [23, 41]. This could suggest a selection of *P. falciparum* to the detriment of other species in the event of symptomatic malaria infection. Another possible explanation is the use of different diagnostic methods. Delicat-Loembet and al. used the 454 sequencing method to identify *Plasmodium* species while other authors used less sensitive methods (PCR and blood smears with RTD) [7, 23, 31]. Furthermore, the low sensitivity of PCR can be explained by an insufficient yield and the low sensitivity of the primer set used. Though we did not report cases of monoinfection with *P. ovale* in our study, this species is sometimes encountered in urban areas and we diagnosed a case of co-infection. In our study, *P. vivax* was not found in any of the children diagnosed as positive, this confirms that *P. vivax* does not circulate in humans in south-eastern Gabon. Furthermore, our analyze highlight a high prevalence of *P. malariae* among asymptomatic children in rural and semi-urban areas, these results are consistent with those of a study conducted in a rural area in Gabon [23, 41]. This can be explained by the section of certain parasitic clones which could be more virulent others, and also by the fact that *P. falciparum* is more apt to induce symptomatology than the other species.

A limitation of this study is that submicroscopic infections were not diagnosed and species were only determined on microscopy positive samples. However, it is important to note that the use of molecular techniques provides more accurate measures of parasite prevalence. These measures suggest more asymptomatic infections.

## Conclusion

To conclude, this study provides the first useful baseline information on malaria prevalence in symptomatic and asymptomatic *Plasmodium* parasite infection **c**hildren, and the *Plasmodium* species circulating in different epidemiological contexts of South-East Gabon. Malaria prevalence is high among children living in rural areas and measures to fight malaria in remote areas of the country should be implemented. Furthermore, asymptomatic infections must be taken into account in order to slow down the transmission of the parasite in the country.

## Data Availability

The datasets analysed in this study are available from the corresponding author on reasonable request.
